# Spectrum of severity of multisystem inflammatory syndrome in children: an EHR-based cohort study from the RECOVER program

**DOI:** 10.1038/s41598-023-47655-y

**Published:** 2023-11-28

**Authors:** Suchitra Rao, Naimin Jing, Xiaokang Liu, Vitaly Lorman, Mitchell Maltenfort, Julia Schuchard, Qiong Wu, Jiayi Tong, Hanieh Razzaghi, Asuncion Mejias, Grace M. Lee, Nathan M. Pajor, Grant S. Schulert, Deepika Thacker, Ravi Jhaveri, Dimitri A. Christakis, L. Charles Bailey, Christopher B. Forrest, Yong Chen

**Affiliations:** 1 Department of Pediatrics, University of Colorado School of Medicine and Children’s Hospital Colorado, 13123 E 16th Ave Box 090, Aurora, CO 80045 USA; 2 Department of Biostatistics, Epidemiology and Informatics, The Perelman School of Medicine, University of Pennsylvania, 423 Guardian Dr, Blockley Hall 602, Philadelphia, PA 19104 USA; 3 Applied Clinical Research Center, Children’s Hospital of Philadelphia, Philadelphia, PA USA; 4 Division of Infectious Diseases, Department of Pediatrics, Nationwide Children’s Hospital and The Ohio State University, Columbus, OH USA; 5 Department of Pediatrics (Infectious Diseases), Stanford University School of Medicine, Stanford, CA USA; 6 Division of Pulmonary Medicine, Cincinnati Children’s Hospital Medical Center and University of Cincinnati College of Medicine, Cincinnati, OH USA; 7 Division of Rheumatology, Cincinnati Children’s Hospital Medical Center and Department of Pediatrics, University of Cincinnati College of Medicine, Cincinnati, OH USA; 8 Division of Cardiology, Nemours Children’s Health, Wilmington, DE USA; 9 Division of Infectious Diseases, Ann & Robert H. Lurie Children’s Hospital of Chicago, Chicago, IL USA; 10 Center for Child Health, Behavior and Development, Seattle Children’s Hospital, Seattle, WA USA; 11 Department of Pediatrics, Perelman School of Medicine, University of Pennsylvania, Philadelphia, PA USA; 12 Present Address: Biostatistics and Research Decision Sciences, Merck & Co., Inc, Kenilworth, NJ USA

**Keywords:** Classification and taxonomy, Epidemiology

## Abstract

Multi-system inflammatory syndrome in children (MIS-C) is a severe post-acute sequela of SARS-CoV-2 infection in children, and there is a critical need to unfold its highly heterogeneous disease patterns. Our objective was to characterize the illness spectrum of MIS-C for improved recognition and management. We conducted a retrospective cohort study using data from March 1, 2020–September 30, 2022, in 8 pediatric medical centers from PEDSnet. We included 1139 children hospitalized with MIS-C and used their demographics, symptoms, conditions, laboratory values, and medications for analyses. We applied heterogeneity-adaptive latent class analyses and identified three latent classes. We further characterized the sociodemographic and clinical characteristics of the latent classes and evaluated their temporal patterns. Class 1 (47.9%) represented children with the most severe presentation, with more admission to the ICU, higher inflammatory markers, hypotension/shock/dehydration, cardiac involvement, acute kidney injury and respiratory involvement. Class 2 (23.3%) represented a moderate presentation, with 4–6 organ systems involved, and some overlapping features with acute COVID-19. Class 3 (28.8%) represented a mild presentation. Our results indicated that MIS-C has a spectrum of clinical severity ranging from mild to severe and the proportion of severe or critical MIS-C decreased over time.

## Introduction

In April 2020, cases of a novel hyperinflammatory disorder associated with SARS-CoV-2 infection were first reported in the UK and Italy^1,2^, which was subsequently termed multisystem inflammatory syndrome in children (MIS-C). The Centers for Disease Control and Prevention (CDC) defines cases as individuals younger than 21 years of age presenting with fever, laboratory evidence of inflammation, and evidence of clinically severe illness requiring hospitalization, with involvement of greater than or equal to 2 organs, with no alternative plausible diagnoses and a positive SARS-CoV-2 test (PCR, serology, antigen) or COVID-19 exposure within the preceding 4 weeks^3^. Reports indicate that most children are 6–12 years of age, of Black/African American race or Hispanic ethnicity, and obesity was the most common pre-existing condition^4–6^. MIS-C is one of the most severe post-acute sequelae of SARS-CoV-2 infection in children, and as of December 2022, the CDC has reported 9370 cases of MIS-C; 76 children have died due to associated complications^7^.

Children with MIS-C can present with a highly heterogeneous patterns of clinical features, making the diagnosis challenging. The clinical presentation can overlap with features of Kawasaki disease, acute COVID-19, toxic shock, and macrophage activation syndrome. Common features include fever, gastrointestinal symptoms (including abdominal pain and diarrhea), cardiac complications (myocarditis and coronary artery dilatation), mucocutaneous findings (rash, mucositis) and respiratory symptoms^8^. Given the wide spectrum of clinical presentations of this novel condition, there is a need to further characterize its disease patterns and their temporal trends. Further elucidation of MIS-C illness spectrum can help identify children at greatest risk of severe outcomes and determine which subgroups may benefit from more targeted therapies.

Most previous research described the symptoms, clinical characteristics and risk factors associated with MIS-C and how these differ from acute COVID-19, Kawasaki Disease and Toxic Shock Syndrome. However, the understanding of subclasses of MIS-C is lacking. One single-center study^9^ of 63 patients conducted in 2020 divided patients into Kawasaki and non-Kawasaki disease subphenotypes. Another CDC study evaluated 3 subclasses of MIS-C in 570 children, with one class representing the highest number of organ systems, a second class with predominant respiratory system involvement, and a third class with features overlapping with Kawasaki Disease. However, this study evaluated cases from March to July 2020, during the early phase of the pandemic when misclassification of cases as Kawasaki disease or acute COVID-19 may have occurred. Therefore, it is not known from the existing literature whether the presentation of MIS-C has changed with newer variants such as delta and omicron.

The objectives of this study were to define the illness spectrum of MIS-C based on symptoms, diagnoses and laboratory parameters and study their temporal dynamics. Our data from PEDSnet provides one of the largest MIS-C cohorts described so far, spanning the entire length of the pandemic, providing sufficient power for detailed analyses.

## Methods

### Data source

This retrospective cohort study is part of the NIH Researching COVID to Enhance Recovery (RECOVER) Initiative, which seeks to understand, treat, and prevent the post-acute sequelae of SARS-CoV-2 infection (PASC). For more information on RECOVER, visit https://recovercovid.org/. The original study cohort has been described elsewhere^10^. We used electronic health record (EHR) data from PEDSnet member institutions^11,12^. Participating institutions included Children’s Hospital of Philadelphia, Cincinnati Children’s Hospital Medical Center, Children’s Hospital Colorado, Ann & Robert H. Lurie Children’s Hospital of Chicago, Nationwide Children’s Hospital, Nemours Children’s Health System (a Delaware and Florida health system), Seattle Children’s Hospital, and Stanford Children’s Health. We retrieved EHR data from all healthcare encounters among hospitalized children and adolescents < 21 years of age who received a COVID-19 diagnosis or vaccination, had a COVID-19 test (PCR, serology or antigen), or demonstrated evidence of respiratory illness or post-acute sequelae of SARS-CoV-2 and had a visit at any of the eight PEDSnet sites between March 2020 and September 2022. From this cohort, we identified encounters with an International Statistical Classification of Diseases, Tenth Revision, Clinical Modification (ICD-10-CM) code for “MIS-C” or “Other specified systemic involvement of connective tissue” from January 1, 2021. Prior to this date, the code “Other specified systemic involvement of connective tissue” was used from March 1, 2020, to December 31, 2020. Data were extracted from the PEDSnet COVID-19 Database-Version 2022-10-20, which included EHR data with dates of service up to September 30, 2022. Cohort entry was defined as the 1 day before the first hospitalization associated with an MIS-C diagnosis term (index hospitalization). We combined hospitalizations if the discharge date of the first hospitalization was within 24 h of the subsequent hospitalization admission date. We excluded thirteen patients with missing admission or discharge dates, as well as patients with Kawasaki Disease during hospitalization.

### Ethical statements

This study constitutes human subject’s research. The study obtained Institute Review Board (IRB) approval under Biomedical Research Alliance of New York (BRANY) protocol #21-08-508. As part of the Biomedical Research Alliance of New York (BRANY IRB) process, the protocol has been reviewed in accordance with the institutional guidelines and regulations. All methods were carried out in accordance with the institutional guidelines and regulations.

The need for informed consent and HIPPA authorization was waived by the Biomedical Research Alliance of New York (BRANY) Institutional Review Board.

### Variables

We included the following indicator variables: demographics, diagnoses (including symptoms and conditions), medications, laboratory values and clinical outcomes (ICU, mechanical ventilation, death), which were coded dichotomously and later grouped to form variables used in our analysis. Conditions and symptoms were identified from ICD-10 or SNOMED code types, including admission and discharge diagnosis codes. The indicator variable was included in the analyses if at least one occurrence was recorded in the EHR from 1 day prior through the entire duration of hospitalization. In addition, age, number of days during hospitalization, number of days in ICU, and laboratory test results were included as continuous or categorical variables.

### Statistical analyses

First, we grouped highly correlated indicator variables, including diagnoses (symptoms and conditions) and laboratory values, into eight broader categories (manifest variables), according to their organ system involvement, which were cardiac, gastrointestinal, hematological, neurologic, renal, respiratory, dermatologic and shock (see Supplementary eTable 1 for definitions of each variable). The variable for a particular organ system was defined based on the occurrence of any symptom, condition, laboratory value, or procedure type related to that system. Less than 10% of patients had missing laboratory test results, which did not affect their classification, since the other indicator variables were highly correlated.

Secondly, using the eight manifest variables as input, we applied a latent class analysis (LCA) model in which an Expectation–Maximization (EM) algorithm was used for estimation^13,14^. To account for heterogeneity across hospitals yet ensuring unified definitions of each latent class, we assumed the same collection of classes but potentially different mixing proportions of each of the subgroups across the health systems. Such generalization of the traditional LCA model is necessary because the prevalence of each subgroup can be substantially different due to different distributions of age, race/ethnicity, and referral patterns which can be strongly associated with the presentation of MIS-C.

For sensitivity analysis, we fitted LCA to the eight manifest variables under different assumed numbers of latent classes, varying from two to eight. Log-likelihood, Akaike Information Criterion (AIC), Bayesian Information Criterion (BIC), entropy, and clinical adjudication among our clinician work group were incorporated to select the appropriate number of clinical-relevant latent classes.

For characterizing the identified latent classes, proportions of the eight manifest variables over the eight broad organ systems across latent classes were displayed as heatmaps of prevalence. To further characterize these identified latent classes, distributions of clinical indicator variables, including demographic characteristics, and individual symptoms, conditions, laboratory results, procedures, and medications, were displayed across the latent classes also using heatmaps of prevalence. To characterize the differences in each continuous clinical variable across latent classes, sample mean, standard error, median and interquartile range were calculated and summarized. The differences of the variables across subphenotypes were tested. To reduce the impact of misclassifications of latent classes, instead of using classification based on maximum posterior probability, all calculations of the characterizations and testing were based on weighted prevalence with weights being the posterior probabilities. The results are presented using heatmaps and forest plots.

To evaluate variation over time, we also evaluated the changes of the proportions for each subphenotype over time in three variant strain periods. Within each time period, the proportions of subgroups were calculated using the average of posterior class membership probabilities. In addition, the change in the prevalence of the eight variables for each of the latent classes over time was estimated.

To further examine the construct validity of our LCA approach, we compared the latent classes of MIS-C to those of COVID-19. We identified a cohort of 183,288 children aged < 21 evaluated from the same institutions in outpatient and inpatient settings between March 1, 2020, and September 30, 2022, who tested positive for COVID-19 by PCR without an MIS-C diagnosis. A description of the cohort has been described previously^10^. We applied LCA to this cohort using the same variables in the MIS-C LCA, evaluated − 7 to 28 days from the first test date. We identified three latent classes of COVID-19. We compared the characteristics of these MIS-C and COVID-19 classes and visualized the distance among them measured by fixation index using multidimensional scaling.

To predict the class membership of a patient, we have provided the formula of posterior class membership probabilities directly derived from LCA, in which the input was the eight manifest variables (shock and seven organ systems) used in LCA, and the output was a vector with each element representing the probability of the patient belonging to the corresponding class. The predicted class membership was the class with the largest posterior probabilities (Supplemental Material). Analyses were conducted using R version 4.1.2 (2021-11-01). The package used for LCA was poLCA 1.4.1 (2014-01-10)^15,16^.

## Results

Among 1,955,736 children in the PEDSnet cohort who received a COVID-19 diagnosis or vaccination, had a COVID-19 test (PCR, serology or antigen), or demonstrated evidence of respiratory illness or post-acute sequelae of SARS-CoV-2 and had a visit at any of the eight PEDSnet sites between March 2020 and September 2022, we identified 1139 children who were hospitalized with MIS-C (Supplementary eFigure 1) without Kawasaki diagnosis during the hospitalization. A description of the study cohort is provided in Table 1. The highest proportion of children (43.3%) were aged between 5–11 years, with a male predominance (61.5%), and non-Hispanic White ethnicity (41.1%). Most (57.9%) children did not have any chronic conditions as defined by the Pediatric Medical Complexity Algorithm (PMCA)^17^. In our MIS-C cohort, 41.4% were admitted to the ICU, 16.1% required mechanical ventilation (invasive and non-invasive), and 15 (1.3%) children died.

**Table 1:** Description of study cohort.

Characteristic	Number of patients (%) (N = 1139)
Age at MIS-C diagnosis, years	
< 1	30 (2.6%)
1–4	230 (20.2%)
5–11	493 (43.3%)
12–15	248 (21.8%)
16–20	138 (12.1%)
Sex	
Female	439 (38.5%)
Male	700 (61.5%)
Race/ethnicity	
Hispanic	235 (20.6%)
Non-Hispanic White	468 (41.1%)
Non-Hispanic Black/African-American	276 (24.2%)
Non-Hispanic Asian/Pacific Islander	43 (3.8%)
Other/unknown	65 (5.7%)
Multiple	468 (41.1%)
Institution	
A	219 (19.2%)
B	108 (9.5%)
C	194 (17.0%)
D	145 (12.7%)
E	191 (16.8%)
F	64 (5.6%)
G	188 (16.5%)
H	30 (2.6%)
Pediatric Medical Complexity Algorithm (PMCA) index	
Non-complex-chronic	660 (57.9%)
Chronic	72 (6.3%)
Complex-chronic	407 (35.7%)
Obesity	135 (11.9%)
Admitted to ICU	472 (41.4%)
Died	15 (1.3%)
Received mechanical ventilation	183 (16.1%)

For mechanical ventilation, we included both invasive (air delivered through intubation) and non-invasive (continuous positive airway pressure, bilevel positive airway pressure, Average Volume-Assured Pressure Support) ventilation. *MIS-C* multisystem inflammatory syndrome in children, *ICU* intensive care unit.

**Figure 1:**
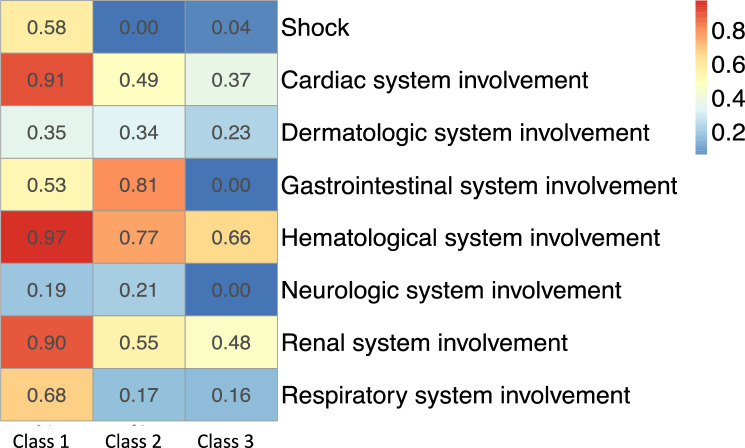
Heatmap of eight organ system variables used in latent class analyses with three classes. The proportions of Class 1, 2, and 3 are 40.0%, 24.0%, and 36.0%, respectively. Each column represents a latent class, and each row represents a manifest variable. The color of the boxes represents the prevalence of variables. The numbers within each cell represents the proportion of patients in a latent class with the manifest variable of interest. The legend on the top right shows the scale of the colors. Red represents prevalence close to 100% and blue represents prevalence close to 0%. The figure was generated using R 4.1.2 with package ‘pheatmap’ 1.0.12^18^ (http://cran.r-project.org/web/packages/pheatmap/).

**Figure 2:**
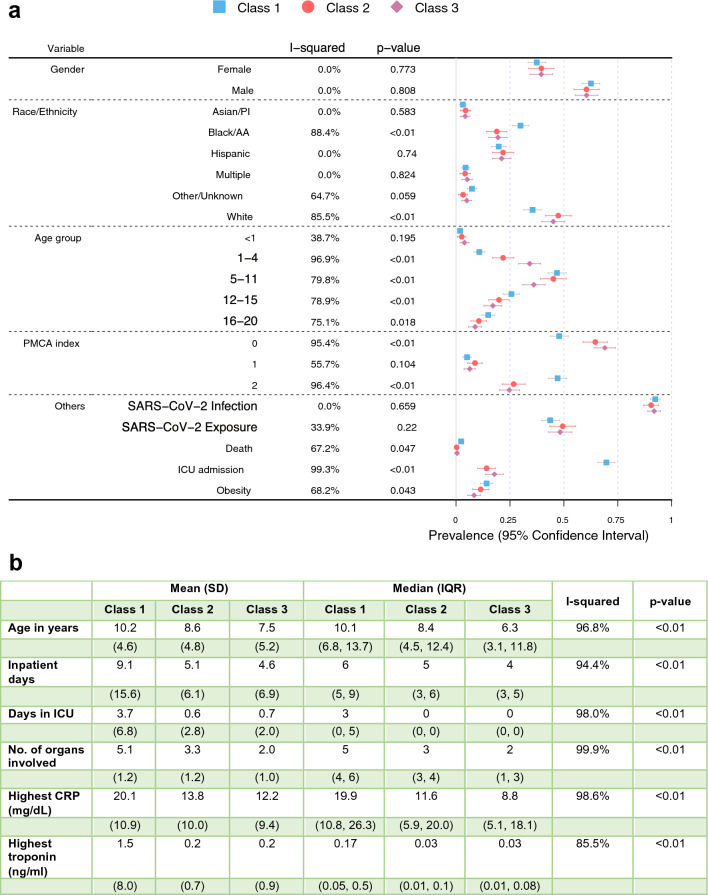
Patient demographic characteristics in three latent classes. (**a**) I-squared, p-value, estimated prevalence, and its 95% confidence interval for discrete variables. Each line plot indicates the estimated prevalence and associated 95% confidence interval of each variable in each class. (**b**) I-squared, p-value, estimated mean, standard deviation, median and interquartile range for continuous variables. The I-squared statistic measures the heterogeneity among the latent classes. p-values are obtained through Cochran’s *Q* test, where the null hypothesis is no between-class heterogeneity. A large I-squared and a small p-squared indicate a large between-class heterogeneity. SARS-CoV-2 infection includes positive PCR, antigen or serology; SARS-CoV-2 exposure indicates that patient had a history of exposure to a contact with known SARS-CoV-2 based on ICD-10 codes; age group is presented in years; Pediatric Medical Complexity Algorithm (PMCA) classified children as complex-chronic (2), chronic (1), or non-complex-chronic (0). *SD* standard deviation, *IQR* interquartile range, *PMCA* Pediatric Medical Complexity Algorithm, *PI* Pacific Islander, *AA* African American, *SARS-CoV-2* severe acute respiratory syndrome coronavirus, *ICU* intensive care unit, *CRP* C-reactive protein.

**Figure 3:**
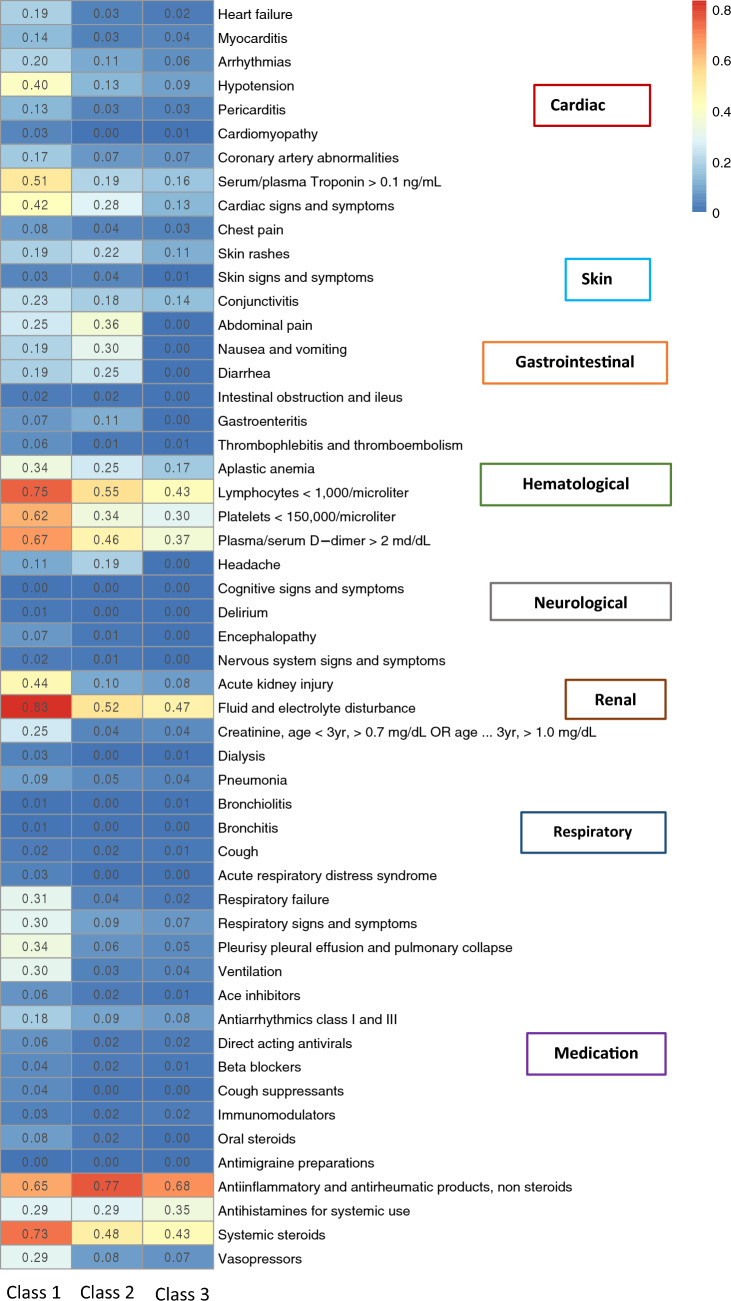
Heatmap of patient characteristics of conditions, laboratory results and medication in three latent classes. Each column represents a latent class, and each row represents a variable. The color of the boxes represents the estimated prevalence of variables. The legend on the top right shows the scale of the colors. Red represents prevalence close to 100% and blue represents prevalence close to 0%. The conditions and laboratory results are ordered based on organ systems. The figure was generated using R 4.1.2 with package ‘pheatmap’ 1.0.12^18^ (http://cran.r-project.org/web/packages/pheatmap/).

**Figure 4:**
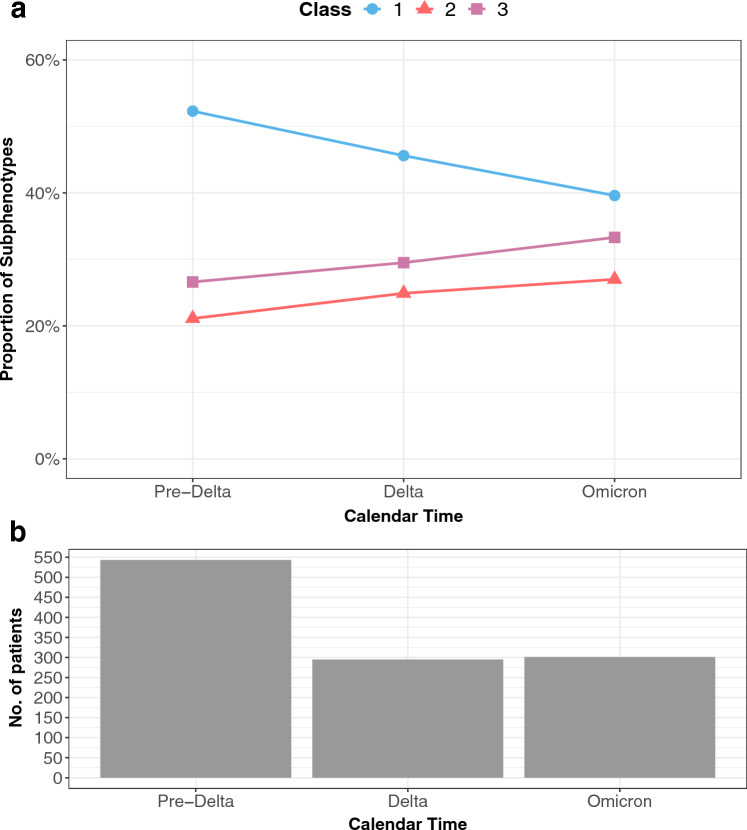
Proportions of three MIS-C classes over time. Each line represents the proportion of the three classes over 3-month periods. The proportions of the classes are calculated using the average of the posterior class membership probabilities of the patients who are diagnosed in the given time window. The calendar time is segmented quarterly. Time intervals with small sample sizes are combined and labeled by a star. (**a**) Proportions of the three latent classes across time. Each line represents a class. (**b**) Histogram of the number of patients in each time window, including timing of predominant SARS-CoV-2 variant activity.

Latent Class analyses identified three subclasses of MIS-C (Fig. 1), with the patient demographics of each class summarized in Fig. 2 and Supplementary eFigure 2. The overall proportions of Class 1, 2, and 3 for the entire period were 47.9%, 23.3%, and 28.8%, respectively. The race and ethnicity of children were similar in each class, with the exception of a higher proportion of children of black/African American race in Class 1. A higher proportion of children 12 years of age and older, and those with a complex chronic condition were identified in Class 1. The proportion of each class by PEDSnet site is shown in Supplementary eTable 2, with some observed heterogeneity. Figure 3 and Supplementary eFigure 3 display specific diagnoses, laboratory results and medications in each group. Class 1 represented children with a more severe clinical phenotype, with 69.7% admitted to the ICU, with a longer mean inpatient stay and longer ICU length of stay. Children in Class 1 had a higher prevalence of cardiac involvement, with abnormal troponin levels, hypotension and shock/dehydration, need for vasopressors and fluid resuscitation. This class was more likely to have abnormal laboratory findings including lymphopenia, elevated d-dimer and thrombocytopenia. There was a higher median number of organ systems involved compared with other classes, with a higher incidence of acute kidney injury, and respiratory involvement (respiratory failure, pleural effusion, need for ventilation). Children in Class 1 had the highest usage of systemic steroids among all classes. Class 2 represented a group with a moderate presentation, with similar multiple organ system involvement to Class 1, including dermatologic (rashes), gastrointestinal (abdominal pain, nausea and vomiting), hematologic abnormalities (lymphopenia, thrombocytopenia, elevated d-dimer), renal (fluid and electrolyte) and respiratory (cardiorespiratory signs and symptoms). Children in Class 2 had a heat map pattern similar to a latent class with acute COVID-19, both with a higher incidence of cardiac and renal system involvement, blood cell abnormalities, and GI system involvement (Supplementary eFigure 4) and 2-dimensional plot demonstrated close proximity between MIS-C Class 2 and COVID-19 Class 1 (Supplementary eFigure 5). This group had the highest use of non-steroidal anti-inflammatory and antirheumatic medications (non-steroidal anti-inflammatory agents represented most medications used in this drug class). Class 3 represented a mild population, with fewer systems involved, and laboratory values demonstrating fluid and electrolyte disturbance, lymphopenia, thrombocytopenia, and elevated d-dimer. All the manifest variables were statistically significantly different across classes with p-values < 0.01. The proportions of each latent class over time are shown in Fig. 4. A higher proportion of children had the more severe MIS-C presentation in the earlier phase of the pandemic (Class 1 represented 52.3% of children during pre-Delta time period), which decreased over time (Class 1 represented 39.6% of children during Omicron). The proportions of all the classes were significantly different over time (p-value < 0.01). Supplementary eFigure 6 shows the prevalence of organ system involvement for patients with MIS-C over time, demonstrating significant declines over time in cardiac, hematological, respiratory involvement, and shock. Patients with gastrointestinal system involvement were more likely to be in Class 2. Latent Class Analyses using 4 and 5 classes are shown in Supplementary eFigure 7 and Supplementary eFigure 8 respectively. The entropy of the model using 3 latent classes was 0.68 (Supplementary eTable 3), and as shown in Supplementary eFigure 9, the posterior class membership probabilities had the highest frequency between 0.9 and 1, indicating distant latent classes.

## Discussion

In our multicenter cohort of 1139 children hospitalized with MIS-C, we identified 3 latent classes with varying illness severity. Class 1 represented the most severe patients, with a higher proportion of children being of age > 12 years, black/African American race, with underlying medical complexity, presenting with hypotension/shock, dehydration, cardiac dysfunction and laboratory abnormalities including lymphopenia, thrombocytopenia, elevated d-dimer and troponin. Class 2 represented an intermediate population with lower illness severity than Class 1 but greater than Class 3, with organ system involvement, including gastrointestinal (nausea, vomiting, abdominal pain, diarrhea), respiratory, hematological and renal, and a lower incidence of cardiac dysfunction and abnormal troponin levels, with a similar pattern to a subset of children with acute COVID-19. Class 3 represented a younger population with the milder form of MIS-C, and fewer organ systems involved (hematological and renal). We also found a higher proportion of patients with the more severe Class 1 phenotype seen earlier in the pandemic, which decreased over time, with lower proportions of children with respiratory, cardiac, and hematological involvement and shock. These findings highlight that MIS-C represents a wide illness spectrum, with variable system involvement. Our findings suggest that the number of organ systems involved, and level of inflammatory marker abnormalities are useful indicators of illness severity, likely reflecting differences in the degree of immune response elicited. The clinical utility of these latent classes based on different levels of severity can help characterize MIS-C through specific early signs/symptoms and laboratory parameters in order to better determine at presentation optimal therapy for these children. Children with milder presentations may warrant different treatment approaches from those with more severe disease, and patients with more severe illness could be identified earlier with closer monitoring and different treatment regimens to improve outcomes.

Our multi-site study included children from diverse geographical regions across the United States, and captured diagnoses, laboratory values, medications, and procedures. Another strength of our study is the use of LCA, which can be especially useful for describing different manifestations of the novel syndrome of MIS-C. It divides patients into groups, based on shared characteristics, allowing for an unbiased determination of phenotypic manifestations^14,19,20^. Importantly, our LCA properly accounts for between-site heterogeneity in patients by allowing different mixing proportions of latent classes, avoiding bias in estimated patient-level class memberships.

Our study is subject to several limitations. Given that inclusion of patients into our cohort was based on a physician’s diagnosis and lack of a gold standard diagnostic test, we may have inadvertently included cases of suspected MIS-C who ultimately had other diagnoses, or children whose illnesses overlapped with Kawasaki disease. Next, our data captures hospitalizations at a PEDSnet site, and data from earlier hospitalizations at other sites was not available. More subtle symptoms from MIS-C may not have been captured in EHR data sources. In addition, we excluded patients with Kawasaki Disease during hospitalization given that they represent distinct entities, acknowledging this approach may exclude some children with MIS-C due to overlapping features of MIS-C and Kawasaki Disease, as well as those in the earlier phases of the pandemic while this syndrome was newly emerging and the ICD-10 code for MIS-C was not yet available. However, we conducted a sensitivity analysis including children with Kawasaki disease, and the identified subphenotypes were similar. Finally, the number of latent classes in LCA may be difficult to determine, which is a well-known challenge in statistical inference, and may lead to deceptive interpretations that the set of latent classes identified in an analysis represents the actual types of individuals in the population. To account for this, we have applied multiple model selection criteria in selecting the number of latent classes. It is known that the using the model selection criteria alone to select the number of latent classes can lead to biased results^21^. Therefore, we considered both the model selection criteria and the clinical interpretation of the latent classes. The model with three latent classes has the highest entropy (Supplementary eTable 3), indicating the classes were well separated, and were also distinct clinically. Adding more classes resulted in similar latent classes that were not distinct to each other and hard to interpret clinically, see eFigure 7 and eFigure 8 for results of four and five classes. In addition, the differences of AIC and adjusted BIC among 3–6 classes was much smaller than the drop from 2 to 3 classes. The final selected LCA model is a parsimonious model, in the sense of achieving a good fit with a small number of parameters, that describes distinct subgroups, which fits with clinical observations and existing literature^22^.

Our unsupervised learning methods using LCA identified a severe MIS-C subgroup with a greater extent of left ventricular dysfunction leading to shock and need for vasopressors/inotropes. Abnormal cardiac strain has been shown to be associated with increased odds of vasoactive support requirement, duration of vasoactive support, and ICU length of stay^23^. An association has also been shown between the degree of inflammation and cardiac injury severity, in particular CRP, troponin and NT-proBNP^24^, as supported by our data. Our data identified that those in the critically ill group were older and more likely to have an underlying medical condition. In the literature, children affected by MIS-C tend to have fewer underlying medical conditions, with obesity a common diagnosis if chronic conditions were present^25^. Older age has been associated with worse outcomes from MIS-C^26^, but the underlying immunologic mechanisms behind this observation have not yet been elucidated.

We identified a latent class with a median of 3 organ systems involved (Class 2), with some similar features with children with acute COVID-19. A similar subphenotype has been described in earlier studies of MIS-C^22^. These and other studies indicate the clinical overlap in some of the presentations of MIS-C and acute COVID-19. Further, the presence of abnormal strain on echocardiogram has also been associated with greater number of organ systems involved^27^, which has been supported by our findings. Our third latent class had a milder presentation, with shorter length of stay and a lower incidence of cardiac involvement. This milder subgroup is an important cohort that warrants further exploration, as they may not require as aggressive treatment with immunomodulators to achieve the same positive outcomes. Further, Classes 2 and 3 had higher non-steroidal anti-inflammatory use than Class 1. One potential explanation for this finding may relate to the higher use of steroids and other immunomodulatory agents in class 1, which may have aborted fever to a greater extent, reducing the need for antipyretic medications.

We evaluated the proportions of each latent class over time and observed a trend towards decreasing frequency of the severe MIS-C class. Earlier reports of MIS-C have indicated high levels of ICU support for patients with MIS-C. In a 2020 systematic review, Ahmed et al. reported on 39 observational studies of MIS-C patients encompassing the period January 1st, 2020, to July 25th, 2020. Out of 662 patients, 71% required admission to the intensive care unit (ICU)^28^. Further exploration of our cohort identified decreasing respiratory system involvement, cardiac involvement and shock, indicating decreased need for critical care interventions. The finding of decreased illness severity of MIS-C during the omicron variant period compared with the alpha and delta periods has been corroborated in other studies^29,30^. The reasons for this decreasing illness severity over time are likely multifactorial. As clinicians’ experience with this new condition matures, earlier identification and treatment may lead to improved outcomes and decreased need for critical care. SARS-CoV-2 immunity in children has been increasing through natural infection and vaccination, rendering fewer children susceptible to first-time encounters with SARS-CoV-2 infection triggering MIS-C. Another hypothesis is that newer SARS-CoV-2 strains are less likely to stimulate the immune response which causes multi-system inflammation.

In conclusion, we identified a clinical spectrum of MIS-C with varying illness severity, sociodemographic characteristics and organ system involvement using latent class analyses. These findings highlight the need for clinicians to recognize the varied clinical spectrum of presentations of MIS-C, and the importance of laboratory parameters to facilitate early evaluation, diagnosis and treatment.

### Supplementary Information

Supplementary Information.

## Data Availability

The data is not publicly available due to privacy concerns. The individual de-identified participant data will not be shared. The data that support the findings of this study may be available through request and DUA process to the corresponding authors.
